# Thrust Versus Non-Thrust Chuna Manipulative Therapy for Low-Back Pain with Pelvic Deviation: A Multicenter Feasibility Randomized Controlled Trial

**DOI:** 10.3390/healthcare14172788

**Published:** 2026-09-01

**Authors:** Yeong-Jae Shin, Sun-Young Park, In-Hyuk Ha, Jun-Su Jang, Mi Hong Yim, Boncho Ku, Sanghun Lee, Hae Sun Suh, Yeon-Woo Lee, In Heo, Man-Suk Hwang, Eui-Hyoung Hwang, Byung-Cheul Shin

**Affiliations:** 1 Department of Korean Medicine Rehabilitation, Spine and Joint Center, Pusan National University Korean Medicine Hospital, Yangsan 50612, Republic of Korea; shinyj5107@gmail.com (Y.-J.S.); lyw2667@gmail.com (Y.-W.L.); drheoin@pusan.ac.kr (I.H.); hwangmansuk@pusan.ac.kr (M.-S.H.); taichi20@daum.net (E.-H.H.); 2 Department of Korean Medicine, School of Korean Medicine, Pusan National University, Yangsan 50612, Republic of Korea; shl0305@gmail.com; 3 Jaseng Spine and Joint Research Institute, Jaseng Medical Foundation, Seoul 07999, Republic of Korea; hanihata@gmail.com; 4 Digital Health Research Division, Korea Institute of Oriental Medicine, Daejeon 34054, Republic of Korea; junsu.jang@kiom.re.kr (J.-S.J.); mh.yim@kiom.re.kr (M.H.Y.); secondmoon@kiom.re.kr (B.K.); 5 Korean Medicine Data Division, Korea Institute of Oriental Medicine, Daejeon 34054, Republic of Korea; ezhani@kiom.re.kr; 6 College of Pharmacy, Kyung Hee University, Seoul 02447, Republic of Korea; haesun.suh@khu.ac.kr; 7 Department of Regulatory Science, Graduate School, Kyung Hee University, Seoul 02447, Republic of Korea; 8 Institute of Regulatory Innovation through Science, Kyung Hee University, Seoul 02447, Republic of Korea

**Keywords:** Chuna manipulative therapy, low-back pain, pelvic deviation, feasibility, randomized controlled trial

## Abstract

**Background/Objectives**: Low-back pain (LBP) is a common musculoskeletal disorder, with a lifetime prevalence reported to be as high as 84%. Chuna manipulative therapy (CMT), a Korean style of manual therapy for correcting joint misalignment, has rarely been evaluated in randomized controlled trials (RCTs). This study primarily aimed to evaluate the feasibility of a future large-scale, multicenter RCT comparing thrust and non-thrust CMT; preliminary clinical and safety data were collected as secondary, exploratory objectives. **Methods**: This multicenter, assessor-blinded pilot RCT randomized 30 participants with non-acute LBP and pelvic deviation into thrust (*n* = 15) or non-thrust (*n* = 15) CMT groups for a 4-week treatment. Feasibility outcomes (eligibility, recruitment, adherence, retention, data completeness, and treatment fidelity) were the primary endpoints. Preliminary clinical effects (Numeric Rating Scale (NRS), Oswestry Disability Index (ODI)) and adverse events were assessed over 24 weeks as exploratory outcomes. **Results**: Of the 35 screened, 30 (85.7%) were randomized, and the planned 15 per center was achieved at both sites. Twenty-nine (96.7%) received at least the minimum number of sessions and were retained to week 24, with complete outcome data and no protocol deviations in intervention delivery. In exploratory analyses, both groups improved from baseline and between-group differences favored thrust CMT, but as the trial was not powered to compare effectiveness, these findings are preliminary only. Nine mild adverse events resolved spontaneously. **Conclusions**: A large-scale, multicenter RCT comparing thrust and non-thrust CMT in pelvic deviation is feasible. The observed feasibility outcomes can inform its progression criteria, whereas the exploratory clinical findings require confirmation in an adequately powered definitive trial.

## 1. Introduction

Low-back pain (LBP) is one of the most common musculoskeletal disorders, with a lifetime prevalence reported to be as high as 84% [1]. Beyond impairing physical function, LBP frequently becomes chronic, and persistent pain substantially diminishes patients’ quality of life (QOL) [2]. Although a range of treatments is available, their efficacy remains inconsistent across patients, underscoring the need for more effective and better-targeted management strategies.

Pelvic alignment relates to LBP at three distinct levels that are often conflated. Anatomical variation reflects structural differences such as innominate morphology or leg length inequality. Functional asymmetry refers to positional or movement asymmetry frequently observed in asymptomatic individuals. Clinically meaningful pelvic deviation is that in which asymmetry is accompanied by symptoms and serves as a direct target for manual intervention. Pelvic asymmetry occurs across the frontal, sagittal, and transverse planes, and has been consistently linked to LBP [3]. Specifically, pelvic tilt is significantly greater in individuals with LBP than in healthy controls, although reported effect sizes are small [4], and asymmetry in pelvic angle and posterior superior iliac spine height is associated with chronic non-specific LBP [5].

This symptomatic form of pelvic deviation is accordingly a routine target of manual therapy, based on the rationale that asymmetric loading on the lumbosacral and sacroiliac joints drives unilateral muscle hypertonicity and compromises lumbar stability [6]. Consequently, manual therapy, including spinal manipulation and mobilization, is recommended for LBP associated with pelvic asymmetry, with the correction of pelvic deviation serving as a primary strategy to restore biomechanical function and relieve pain.

Among the manual therapies used for this purpose, Chuna manipulative therapy (CMT) is a nonpharmacological traditional Korean medicine intervention that applies manipulation to the spine and pelvis to correct joint misalignment and restore physiological function [7]. While traditionally conceptualized through structural balance and meridian theories, modern clinical applications of CMT align with neurophysiological and biomechanical principles of manual therapy. CMT is thought to improve joint mobility, reduce paraspinal muscle hypertonicity, and enhance postural stability by integrating musculoskeletal physiology with functional restoration principles [8], and is increasingly being recognized for managing musculoskeletal disorders, such as LBP. Indeed, with LBP projected to affect over 800 million people worldwide by 2050, recent international guidelines, including that of the World Health Organization (WHO), now recommend non-pharmacological and manual therapies as first-line care [9,10]. In line with this global trend, CMT use in South Korea grew markedly following national health insurance coverage in April 2019, from 3.17 million sessions in 2019 to 4.79 million in 2023 [11].

Under Korean national health insurance, CMT is reimbursed differently depending on whether complex (thrust) or simple (non-thrust) techniques are used. Complex CMT requires the presence of joint dysfunction, whereas simple CMT does not. Because pelvic deviation constitutes such a joint dysfunction [12], both techniques may be applied in these patients. Their comparative effectiveness in this population is therefore a policy question as well as a clinical one. Previous comparisons of thrust and non-thrust techniques have reported generally comparable outcomes [13], but were not designed with the correction of pelvic asymmetry as their objective. For CMT specifically, no comparison has been conducted in a population selected on the basis of pelvic deviation, and long-term follow-up beyond the treatment period has rarely been reported.

Addressing this gap would require a large-scale confirmatory randomized controlled trial (RCT). However, the feasibility of delivering such a trial must be established beforehand, both to improve the likelihood that the definitive trial succeeds and to address budgetary and safety considerations [14]. Therefore, the primary aim of this study was to assess the feasibility of a future multicenter RCT in terms of eligibility, recruitment, adherence, retention, data completeness, and treatment fidelity. Preliminary clinical and safety data were collected as a secondary, exploratory objective, to inform the feasibility and design of the definitive trial rather than to test comparative effectiveness.

## 2. Materials and Methods

### 2.1. Study Design

This study was a two-armed parallel, multicenter, assessor-blinded pilot RCT conducted at Pusan National University Korean Medicine Hospital (Yangsan, Korea) and Jaseng Korean Medicine Hospital (Seoul, Korea).

Participants who met the inclusion criteria through a screening process were randomized into either the thrust or non-thrust CMT group. Treatments were administered spanning 4 weeks (weeks 1–4), followed by follow-up assessments at week 5, week 12, and week 24 (i.e., 1, 8, and 20 weeks after treatment completion) (Figure S1 and Table S1). Participants were enrolled for treatment within 3 days of screening and received treatment 3–4 times a week for 4 weeks. The overall study period spanned from 30 May 2022 to 1 June 2023, from enrollment of the first participant to completion of the final follow-up assessment.

Data management and monitoring were performed regularly by a CRO at the two institutions, ensuring data reliability and consistency in the research process. In addition to assessing the comparative effectiveness and safety of CMT, this trial was designed to include an economic evaluation, led by Prof. Hae Sun Suh (College of Pharmacy, Kyung Hee University, Seoul, Korea). The findings of this economic evaluation will be reported separately.

### 2.2. Trial Adherence to Guidelines and Ethical Considerations

**Figure 1:**
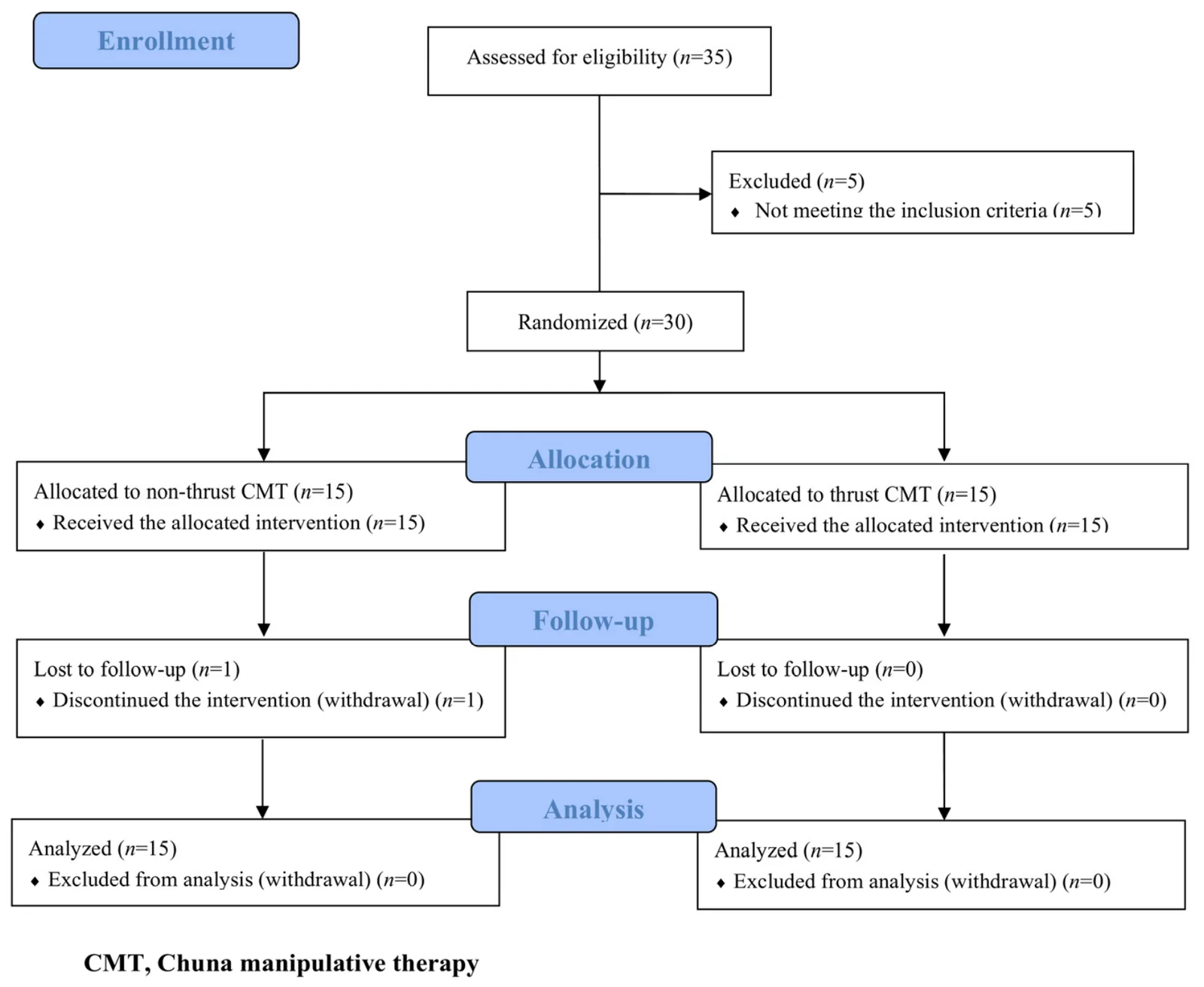
Flow chart of the study.

This study was reported in accordance with the Consolidated Standards of Reporting Trials (CONSORT) 2025 statement [15]. The detailed research procedures are summarized in Figure 1. This trial was prospectively registered with the Clinical Research Information Service (CRIS) before the enrollment of the first participant (https://cris.nih.go.kr; KCT0007090; registered on 17 March 2022). This study was approved by the Institutional Review Board (IRB) of Pusan National University Korean Medicine Hospital (PNUKHIRB-2021-12-001) and Jaseng Korean Medicine Hospital (JASENG-2021-12-014-003) prior to the recruitment of any participants.

### 2.3. Participants

Recruitment was conducted after publicizing through hospital posters, homepage advertisement, and direct communication with participants. All participants provided written informed consent prior to enrollment.

#### 2.3.1. Inclusion Criteria

Participants were enrolled if they met the following eligibility criteria: (i) non-acute LBP (onset > 2 weeks) accompanied by pelvic deviation, with a pain intensity score of ≥5 on the Numeric Rating Scale (NRS); (ii) evidence of pelvic deviation confirmed by manual diagnosis with X-ray imaging; (iii) age between 19 and 65 years; (iv) capable of normal communication; and (v) voluntary participation in the study, as per the signed informed consent form.

Pelvic deviation was assessed independently by two Korean medicine doctors at each site, both of whom had completed SOP training before the start of the trial. Manual examination comprised a functional leg-length analysis test, in which side-to-side differences in leg length were assessed in the supine position, and a sitting posture flexion test, in which asymmetry in the movement of the posterior superior iliac spines (PSIS) was observed during forward flexion. Only participants judged positive on manual examination underwent radiography, and a standing anteroposterior pelvic radiograph was obtained on the same day using a digital radiography system (Aristos MX; Siemens Healthineers, Erlangen, Germany) and measured on a picture archiving and communication system (PACS). Classification followed quantitative criteria prespecified in the trial SOP, based on side-to-side differences rather than absolute values. Iliac rotation was determined primarily from the innominate measurement, and inflare and outflare from the ilium shadow measurement; femoral head height, obturator foramen area, off-centering relative to the pubic symphysis, and the S2–PSIS distance served as supporting criteria. Where the two assessors reached different conclusions, the case was resolved by consensus, and participants were enrolled only when pelvic deviation was confirmed on both manual examination and radiography.

Radiographic findings were recorded as classification outcomes against the SOP criteria rather than as continuous measurements. Because eligibility required confirmed pelvic deviation, the enrolled sample contained no negative cases, and inter-assessor agreement could not be quantified from these data.

The >2-week criterion was applied to exclude patients in the acute phase, in whom spontaneous recovery is frequent and manual treatment requires additional caution, rather than to define a duration-based LBP phenotype; the target population of this trial was defined by the presence of pelvic deviation.

#### 2.3.2. Exclusion Criteria

Participants were excluded from the study if they met any of the following criteria: (i) diagnosis of severe specific diseases that could cause LBP (e.g., spinal metastasis of tumors, acute fractures, or poliomyelitis) or in which LBP was not attributed to pelvic deviation; (ii) absolute contraindications to CMT (e.g., acute cauda equina syndrome, spinal dislocation, or aneurysm); (iii) deemed ineligible for trial by the clinical research team owing to a history of lumbosacral or pelvic surgery, knee surgery, congenital spinal disorders, or nerve block procedures; (iv) having received CMT within the past week or currently using medications that could affect study outcomes, including steroids, immunosuppressants, psychiatric drugs, or nonsteroidal anti-inflammatory drugs (NSAIDs); (v) being scheduled for surgeries or treatments during the study period that could influence the study outcomes; (vi) diagnosis of chronic diseases (e.g., cardiovascular diseases, renal diseases, diabetic neuropathy, dementia, and epilepsy) that could impact the study results; (vii) participation in another clinical trial within the past month; (viii) inability to effectively communicate due to dementia or mild cognitive impairment; (ix) pregnant women; and (x) inability to complete the informed consent process or deemed unsuitable subject to other reasons.

### 2.4. Randomization and Blinding

Randomization lists were generated by an independent statistician (Yim MH) using block randomization (at a 1:1 ratio) with the SAS software (version 9.4, SAS Institute Inc., Cary, NC, USA). Randomization was implemented independently at each site to ensure no overlap of randomized numbers. These lists were securely managed, and participants were sequentially allocated using sequentially numbered, sealed, opaque envelopes.

Owing to the nature of the CMT intervention, blinding of practitioners and participants was not feasible. However, to minimize bias, assessors were blinded to group allocations and were not involved in the intervention or randomization process. All assessments were carried out independently in a separate room to ensure impartiality, and the results were recorded in electronic case report forms (eCRFs).

### 2.5. Sample Size Calculation

As this was a pilot study intended to test the feasibility of a larger trial, the sample size was not based on a formal power calculation, in line with established recommendations for pilot and feasibility studies. The target was set at 12 analyzable participants per group (24 in total); accounting for an anticipated 15% dropout rate, this was inflated to 15 participants per group (30 in total; thrust CMT *n* = 15 vs. non-thrust CMT *n* = 15).

### 2.6. Interventions

Chuna manipulative therapy (CMT) is a Korean manual therapy comparable to Western spinal manipulation and mobilization, performed by hand on the spine and pelvis. All CMT treatments were based on standard Korean medicine practices and administered by licensed Korean medicine doctors (KMDs) having at least 3 years of clinical experience. All practitioners underwent standardized operating procedure (SOP) training conducted by the principal researcher prior to the study. This training ensured consistency in the application protocols across practitioners and sites. The two interventions compared in this study are described below.

#### 2.6.1. Thrust CMT

Thrust CMT employs high-velocity, low-amplitude (HVLA) techniques that extend beyond the physiological range of joint motion, thereby increasing the joint space and restoring physiological mobility [12]. In accordance with the national definition of complex CMT, each session comprised non-thrust preparatory techniques to relax the target region, followed by an HVLA adjustment directed at the segment identified as deviated. The target segment and technique were determined by the treating KMD according to the pelvic deviation pattern identified at diagnosis; representative techniques, with the corresponding indication, patient position, and direction of the corrective force, are presented in Table S2. Each session lasted approximately 15 min and was administered by the KMDs following the SOP.

#### 2.6.2. Non-Thrust CMT

Non-thrust CMT involves movements within the physiological range of joint motion to stimulate the muscles, fascia, and soft tissues, comprising joint mobilization, joint distraction, and myofascial release [12,16]; no HVLA thrust was applied in this arm. At each session the treating KMD applied at least one technique, as prespecified in the protocol (Table S2). Each session lasted approximately 15 min and was administered by the KMDs following the SOP.

#### 2.6.3. Concomitant Medications and Therapies

Medications taken at least 4 weeks prior to the study initiation could be continued at the discretion of the clinical researcher, provided they were not contraindicated (e.g., antihypertensive medications). Medications and treatments that could affect symptoms were not permitted during the clinical trial. However, under urgent circumstances, minimal rescue medication was allowed at the discretion of the clinical researcher. Prohibited medications and treatments included corticosteroids, NSAIDs, antipsychotics, antidepressants, benzodiazepines, hypnotics (unless administered at a stable dosage before the study), antibiotics, and LBP-related interventions such as physical therapy, herbal medicine, and acupuncture. All concomitant medications were documented in the participants’ eCRFs, including details, such as the medications’ name, dosage, purpose, and duration of use.

#### 2.6.4. Intervention Fidelity Procedures

Beyond the initial SOP training, several procedures were used to maintain intervention fidelity. The techniques performed were recorded in the eCRF at every session, allowing adherence to the prespecified technique sets to be verified rather than assumed. Trial conduct was monitored throughout the study period by an independent monitor from a contract research organization (Korea Institute of Oriental Medicine), who reviewed study progress by telephone, e-mail, and on-site visits and verified eCRF entries against source documents. Data quality control followed the trial SOP, under which corrections to the eCRF were made in a traceable manner with the date, reason, and signature of the person making the change.

### 2.7. Outcome Measures

#### 2.7.1. Feasibility Outcomes

Feasibility was assessed using six process outcomes. The trial protocol had specified recruitment together with a combined retention and adherence measure; in this report these are separated and defined as follows.

(i) Eligibility rate: the proportion of screened individuals who met all eligibility criteria. (ii) Recruitment rate: the number of participants randomized per month at each center over the recruitment period. (iii) Adherence: the proportion of randomized participants who received at least the protocol-specified minimum of eight treatment sessions, and the number who completed the full planned course of 12 sessions. (iv) Retention: the proportion of randomized participants who completed the week 24 follow-up assessment. (v) Data completeness: the proportion of expected outcome data that were available across assessment points. (vi) Treatment fidelity: protocol deviations relating to intervention delivery recorded during monitoring.

Progression criteria were not prespecified in the protocol; the observed values are therefore used to propose thresholds for the definitive trial.

#### 2.7.2. Preliminary Clinical Outcomes

LBP severity was evaluated using the NRS, a standardized tool used to quantify pain intensity. Participants rated their pain on a scale from 0 (no pain) to 10 (worst imaginable pain) based on its intensity experienced over the prior week [17].

Baseline was the measurement obtained before the first treatment at week 1. Thereafter, the NRS was assessed once weekly at the first visit of each treatment week, immediately after treatment (weeks 1–4). Because the NRS refers to pain intensity over the preceding week, weekly assessment ensured that the recall periods of successive measurements did not overlap. Follow-up assessments were performed at week 5, week 12, and week 24, corresponding to 1, 8, and 20 weeks after the end of treatment, with no treatment administered at those visits.

The Oswestry Disability Index (ODI) is validated to assess functional disability during daily activities. It consists of 10 items, each scored on a scale of 0–5, with a maximum total score of 50, where higher scores indicate greater levels of disability. This study used the Korean version of the ODI, which has been validated for reliability and accuracy [18]. Baseline was obtained before the first treatment at week 1, and follow-up assessments were performed at week 5 and week 12, corresponding to 1 and 8 weeks after the end of treatment.

The Patients’ Global Impression of Change (PGIC) is a subjective 7-point scale used to evaluate the overall improvement perceived by patients following treatment. The scale ranges from 1 (very much improved) to 7 (very much worse), with lower scores indicating greater improvement [19]. The PGIC was assessed once, at week 5, corresponding to 1 week after the end of treatment.

Pain intensity was selected as the main clinical indicator to monitor long-term trends and was followed to week 24, whereas the ODI was assessed to week 12 and the PGIC at week 5. This schedule was chosen to limit participant burden in a pilot trial in which retention was itself a feasibility outcome; the PGIC was administered once, immediately after the treatment period, as it was intended to capture the participant’s overall impression of change attributable to that period.

The Credibility and Expectancy Questionnaire evaluated participants’ expectations and confidence in the treatment’s efficacy [20]. At the first treatment visit in week 1 (visit 2), participants responded to the question, “How much do you expect this treatment to alleviate your symptoms?” Responses were rated on a 9-point Likert scale ranging from 1 (not at all) to 9 (very much).

### 2.8. Economic Evaluation

To assess health-related QOL for economic evaluation, the EQ-5D-5L and EQ-VAS were measured at baseline, after each weekly treatment, and at week 5, week 12, and week 24, corresponding to 1, 8, and 20 weeks after the end of treatment. The SF-12v2 was assessed at week 5 and week 12, corresponding to 1 and 8 weeks after the end of treatment. Cost data, including direct medical and non-medical costs and indirect costs, were obtained throughout the trial. A comprehensive economic analysis based on these data, conducted by a health economics expert (Suh HS), will be reported in a separate publication.

### 2.9. Adverse Events (AEs)

Unexpected symptoms, signs, or diseases observed during the clinical trial, regardless of their association with the CMT intervention, were defined as AEs. AEs were collected through participants’ self-reports and observations by clinical researchers. The association between AEs and CMT interventions was assessed by a trained clinical researcher using a six-point classification system (1 = definitely related, 2 = probably related, 3 = possibly related, 4 = probably not related, 5 = definitely not related, and 6 = unknown). AEs with a score of 1 to 3 were considered to have a potential association with the treatment. The severity of AEs was evaluated using established criteria, such as the WHO Adverse Reaction Severity Grading System [21] or, if unsuitable, Spilker’s 3-tier classification system [22].

Clinical trial coordinators at each participating institution educated the participants and their guardians on the potential for AEs and instructed them to report any occurrences immediately. Details regarding AEs, including type, time of occurrence, severity, treatment, progression, and association with the intervention, were documented in the eCRFs and preserved in compliance with clinical trial management standards.

In the event of a serious AE (SAE), it was reported to the IRB of the respective institution and the lead clinical trial center (Pusan National University Korean Medicine Hospital) to decide whether to continue or terminate the study.

### 2.10. Termination Rules

The trial completion status and reasons for withdrawal or dropout were clearly documented for all participants, and withdrawn participants were not replaced.

Participants were discontinued from the trial for the following reasons: (i) occurrence of an SAE, potential harm, or other medical reasons; (ii) voluntary withdrawal of consent; (iii) protocol violation (e.g., failure to meet the minimum 8 treatment sessions); or (iv) inability to follow-up.

The entire study could be terminated prematurely by the principal researcher in consultation with the sponsoring institution under the following circumstances: (i) identification of unanticipated severe or unacceptable risks to the participants; (ii) occurrence of moderate SAEs related to the intervention in more than 25% of the participants; or (iii) failure to recruit the target number of participants within the allotted period.

### 2.11. Statistical Analysis

All analyses were based on the full analysis set (FAS), which comprised all 30 randomized participants according to the intention-to-treat principle.

The preliminary clinical outcomes (NRS and ODI) were analyzed using linear mixed-effects models fitted to all observed measurements, including the baseline visit. Each model included group, visit, the group-by-visit interaction, a baseline-by-visit interaction, and center (two sites) as fixed effects, together with a participant-level random intercept; the random intercept induces a compound-symmetry within-subject covariance structure in which the total variance is constant across visits and any two visits from the same participant share a common correlation. Because the baseline visit was retained in the response, the model yields a baseline estimated marginal mean (EMM) that is common to both groups by construction. Center was modeled as a fixed rather than a random effect, consistent with the center-stratified randomization, because a variance component based on only two centers cannot be estimated reliably; inference is therefore conditional on the participating centers. Models were estimated by restricted maximum likelihood (REML), and denominator degrees of freedom were obtained using the Kenward and Roger approximation.

The trial protocol prespecified that missing values would be analyzed as observed, without imputation. The observed-data likelihood of the linear mixed-effects model is consistent with this prespecified approach: missing outcome values were not imputed, and all observed data contributed to the likelihood, which provides valid inference under the missing-at-random assumption. The protocol also allowed adjustment for covariates influencing the outcome, and baseline values and center were included accordingly. Between-group differences (thrust minus non-thrust CMT) at each visit and within-group changes from baseline were estimated as model-based contrasts of the EMMs; within-group changes from baseline were reported with Dunnett-adjusted *p*-values, whereas between-group differences were reported without adjustment. Standardized effect sizes (Cohen’s *d*) were derived from the corresponding t statistics. Consistent with the exploratory nature of this feasibility study, emphasis was placed on effect estimates with 95% confidence intervals rather than on *p*-values alone.

Descriptive statistics, including the mean (or median), standard deviation (or interquartile range), confidence intervals, minimum, and maximum, were calculated for continuous variables. Frequencies and percentages were calculated for categorical variables, which were compared between groups using the chi-squared test or Fisher’s exact test; baseline continuous characteristics were compared using the two-sample *t*-test or Wilcoxon rank-sum test, as appropriate. The PGIC, which was assessed only once at week 5, was not amenable to longitudinal modeling and is therefore reported descriptively.

All statistical analyses were performed in R version 4.6.0 (R Foundation for Statistical Computing, Vienna, Austria), with the linear mixed-effects models fitted using the lme4 package, at a two-sided significance level of 0.05.

## 3. Results

### 3.1. Participants

Figure 1 presents the CONSORT flow diagram detailing participant enrollment, randomization, follow-up, and analysis processes. All 30 randomized participants were included in the analysis (FAS). Table 1 summarizes the baseline demographic characteristics of the participants. The two groups exhibited no statistically significant differences.

### 3.2. Feasibility Outcomes

Feasibility outcomes are reported separately below.

(i) Eligibility rate: Of 35 individuals screened, 30 (85.7%) met all eligibility criteria and were randomized; five were excluded for not meeting the inclusion criteria.
(ii) Recruitment rate: Participants were enrolled between 30 May 2022 and 23 December 2022, a recruitment period of 6.8 months, giving an overall rate of 4.4 participants randomized per month. At Pusan National University Korean Medicine Hospital, 15 participants were randomized over 4.4 months (3.4 per month), and at Jaseng Korean Medicine Hospital, 15 participants over 2.0 months (7.5 per month). The two centers began enrollment at different times, and the planned allocation of 15 participants per center was achieved at both sites.
(iii) Adherence: Twenty-nine of the 30 randomized participants (96.7%) received at least the protocol-specified minimum of eight treatment sessions. Of these, 27 completed all 12 planned sessions, one received 11, and one received 10. The participant who withdrew consent during the treatment period had received three sessions.
(iv) Retention: Twenty-nine participants (96.7%) completed the week 24 follow-up assessment.
(v) Data completeness: All scheduled outcome data were obtained from the 29 participants who completed the trial; the participant who withdrew during the treatment period contributed data up to week 1.
(vi) Treatment fidelity: No protocol deviations relating to the delivery of the allocated intervention were recorded during monitoring. Protocol deviations were recorded for two participants whose visits fell outside the permitted visit window, owing to COVID-19-related restrictions or personal circumstances; in both cases the allocated intervention was delivered as specified. In addition, no participant required rescue medication or received prohibited concomitant care during the trial period.

### 3.3. Preliminary Clinical Outcomes

**Figure 2:**
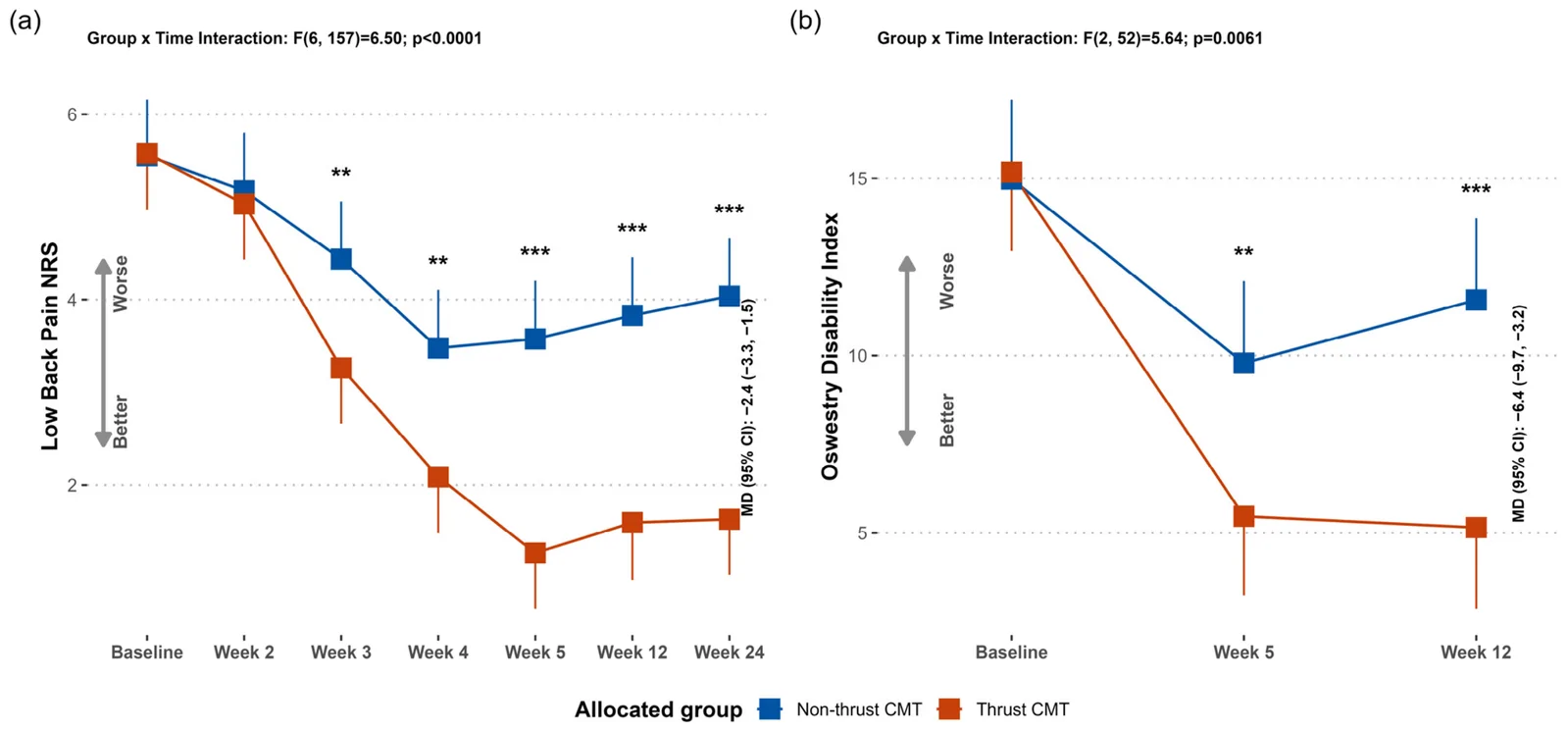
Model-based estimated marginal means (95% CI) by group across visits. (**a**) low-back pain NRS; (**b**) Oswestry Disability Index. One-sided CI whiskers are shown (non-thrust CMT upward, thrust CMT downward). MD, mean difference (Thrust−Non-thrust CMT) at the final visit. Asterisks denote the significance of the between-group difference at each visit (** *p* < 0.01, *** *p* < 0.001).

The preliminary clinical outcomes are summarized as model-based estimates in Table 2, with the corresponding joint tests in Table 3 and the estimated trajectories in Figure 2. Consistent with the exploratory aim of this feasibility study, these between-group comparisons are reported as preliminary, hypothesis-generating signals rather than as evidence of comparative effectiveness.

For LBP NRS, pain decreased from baseline in both groups. The group-by-time interaction was significant (*F*(6, 157) = 6.50, *p* < 0.001), indicating that the two groups followed different trajectories over the study period. Model-based estimated marginal means (EMMs) were similar at baseline and through week 2, after which they diverged in favor of thrust CMT. The between-group difference (thrust minus non-thrust CMT) reached statistical significance from week 3 onwards and was largest in the treatment period: the mean difference (MD) was −1.4 (95% CI: −2.3, −0.5; Cohen’s *d* = −0.51) at week 4 and −2.3 (−3.2, −1.4; d = −0.85) at week 5. This separation was maintained through the follow-up period, with an MD of −2.2 (−3.1, −1.3; d = −0.80) at week 12 and −2.4 (−3.3, −1.5; d = −0.89) at week 24, all favoring thrust CMT (all *p* < 0.001).

For ODI, disability also decreased from baseline in both groups, and the group-by-time interaction was significant (*F*(2, 52) = 5.64, *p* = 0.006). The groups were comparable at baseline, and the between-group difference favored thrust CMT at both post-baseline visits: the MD was −4.3 (95% CI: −7.5, −1.1; Cohen’s *d* = −0.64; *p* = 0.009) at week 5 and −6.4 (−9.7, −3.2; d = −0.93; *p* < 0.001) at week 12.

The PGIC distribution at week 5 favored thrust CMT (*p* < 0.001). In the thrust group, all participants reported improvement, most rating themselves as very much or much improved (14/15, 93.3%). In the non-thrust group, improvement was more modest, with most reporting only minimal improvement (8/14, 57.1%) and 3 (21.4%) reporting no change (Figure S2).

Baseline treatment expectancy did not differ significantly between the groups (*p* = 0.163, Fisher’s exact test), indicating comparable expectations of treatment benefit at the outset. This reduces the likelihood that the observed between-group differences are attributable to differential expectations, although, given the small sample and the exploratory design, these clinical findings require confirmation in an adequately powered trial.

### 3.4. Adverse Events

During the clinical trial, 9 mild AEs were reported according to Spilker’s classification, with 4 in the thrust CMT group and 5 in the non-thrust CMT group. Primary AEs included headache, hip pain, transient pain, indigestion, and COVID-19. Of these, 8 were assessed as unrelated to the treatment, and 1 case (in the non-thrust CMT group) was classified as possibly related. All events were mild, resolved spontaneously without intervention, and none led to dropout or code unblinding. No AEs were classified as probably or definitely related to either intervention.

## 4. Discussion

This multicenter pilot trial assessed whether a definitive randomized trial comparing thrust and non-thrust CMT in patients with LBP and pelvic deviation would be feasible and collected preliminary clinical and safety data to inform its design. Although CMT is widely used for musculoskeletal disorders [23], head-to-head evidence distinguishing these two approaches remains limited, and the distinction carries reimbursement consequences under Korean national health insurance. Establishing whether such a trial can be delivered is therefore a necessary step before comparative effectiveness can be addressed.

Recruitment, retention, and adherence were high across both centers. Both centers reached their planned allocation, almost all participants completed the 24-week follow-up, and those who remained received the treatment course as planned. The single withdrawal occurred early and was unrelated to safety, and the recorded protocol deviations did not affect delivery of the interventions. The trial procedures therefore appear acceptable to participants and deliverable by practitioners across two centers.

These findings inform the design of the definitive trial. The treatment schedule over four weeks can be retained without modification. Two elements require adjustment: First, the number of participating sites and the length of the recruitment period should take account of the center-specific recruitment rates observed here. Second, wider visit windows or remote follow-up options should be adopted, since the protocol deviations recorded in this pilot were confined to visit scheduling rather than to the intervention itself. Three elements should be added to the protocol. First, pelvic alignment should be included as a prespecified outcome and recorded as continuous measurements before and after treatment, so that mechanistic hypotheses can be tested directly rather than inferred. Second, explicit progression criteria should be prespecified, as these were not defined in the present pilot; the values observed here are used instead to propose thresholds for the larger trial. Third, all outcome measures should be assessed to week 24, since disability and global impression of change were followed only to weeks 12 and 5 [24], which left the durability of any functional gain unexamined. The sample size for this full-scale study was derived from a previous multicenter pragmatic trial of CMT for non-acute LBP [25]. The variability of the outcomes observed in this pilot can be used to check and, if necessary, update the standard deviation assumed in that calculation, thereby refining the sample size for the definitive trial.

In this pilot, both interventions were associated with improvement in pain and disability from baseline, and the interaction between group and time was significant for both NRS and ODI, indicating that the two groups followed different trajectories over the study period. The estimated between-group differences consistently favored thrust CMT, emerged during the treatment period, and were maintained through week 24, with moderate to large effect sizes. These estimates should nonetheless be interpreted with caution, as the trial was not powered for comparison between groups, the confidence intervals are wide, and the analyses were exploratory.

A biologically plausible explanation is that manual therapy acts through both biomechanical and neurophysiological pathways, including modulation of nociceptive input and activation of descending inhibitory pathways [26]; this interpretation remains speculative, however, because change in pelvic alignment was not assessed as a prespecified outcome and no objective biomechanical measure was obtained, so any explanation based on the restoration of pelvic balance cannot be tested with the present data. Previous randomized trials of thrust versus non-thrust techniques in mechanical or chronic LBP have generally reported comparable outcomes between the two approaches [27,28], and systematic reviews of manipulation and mobilization have reached broadly similar conclusions [13]. Those studies, however, were not conducted in populations selected for confirmed pelvic deviation, whereas the present pilot targeted this specific subgroup; the between-group differences observed here may therefore reflect a response particular to this population, but given the exploratory design they should be regarded as hypothesis-generating and confirmed in an adequately powered trial.

Adverse events were infrequent and mild in both arms, occurred at similar frequency, and none were classified as probably or definitely related to either intervention. All events resolved spontaneously, and none led to withdrawal. These observations are consistent with the safety profile reported for CMT in large-scale observational data [29], and they indicate that the safety monitoring procedures used here were workable. They do not, however, establish that the two interventions are equally safe: a sample of this size cannot detect differences in the frequency of adverse events, and serious events associated with thrust techniques are too rare to be observed in a trial of 30 participants. Comparative safety will require a much larger sample or dedicated observational data.

Several limitations should be considered. First, as a pilot trial, this study was not powered to detect between-group differences, and all clinical comparisons should be regarded as exploratory. In addition, because of the way complex CMT is defined (Section 2.6.1), participants in the thrust arm received the HVLA component in addition to, rather than instead of, non-thrust treatment. The between-group difference therefore represents the incremental effect of adding an HVLA adjustment rather than the effect of the thrust technique in isolation, although total session duration was equivalent between arms. Second, the assessment of pelvic deviation has inherent limitations. Palpation-based assessment of pelvic landmarks has shown limited inter-examiner agreement in previous studies [30], and inter-assessor agreement was not quantified in the present trial, as the enrolled sample contained only confirmed cases. Pelvic alignment was re-examined at week 5 in accordance with the protocol but was not prespecified as an outcome, so changes after treatment are not reported. No objective biomechanical outcome measures were obtained. Third, although outcome assessors were blinded, neither participants nor practitioners could be blinded, and no sham comparator was used. Baseline treatment expectancy was similar between groups, but participants were necessarily aware of the intervention they received, and the outcomes used are self-reported and therefore susceptible to non-specific treatment effects. Finally, the sample was small and predominantly female, and recruitment was restricted to two Korean medicine hospitals, so practitioner and center effects cannot be excluded and generalizability beyond Korean medicine settings is limited. These limitations are addressed in the design considerations for the definitive trial outlined above.

Taken together, this pilot trial indicates that a multicenter randomized trial comparing thrust and non-thrust CMT in patients with LBP and pelvic deviation can be delivered as designed, and it identifies the parameters and protocol adjustments required for such a trial; the clinical findings reported here are exploratory and await confirmation in that trial.

## 5. Conclusions

A definitive randomized trial comparing thrust and non-thrust CMT in patients with non-acute LBP associated with pelvic deviation appears feasible. Eligibility, recruitment, adherence, retention, data completeness, and treatment fidelity were all high. The recruitment rates and protocol modifications identified here provide the basis for the design of that trial.

Preliminary clinical outcomes favored thrust CMT, but the trial was not powered to test comparative effectiveness. These findings are exploratory and require confirmation in an adequately powered trial.

## Figures and Tables

**Table 1:** Baseline demographic characteristics and health status variables of the participants.

Variables Mean (95% CI) or *n* (%)	Group		*p*-Value ^†^	*p*-Value ^‡^
	Non-Thrust CMT (*n* = 15)	Thrust CMT (*n* = 15)		
Age (years)	53.6 (48.8, 58.4)	49.5 (43.0, 55.9)	0.280	0.309
Sex				
Male	3 (20.0)	2 (13.3)		
Female	12 (80.0)	13 (86.7)		
BMI (kg/m^2)^	22.2 (21.2, 23.1)	24.3 (22.2, 26.4)	0.061	0.135
Height (cm)	162.6 (157.8, 167.5)	159.2 (155.9, 162.6)	0.231	0.253
Weight (kg)	58.9 (53.8, 64.1)	61.8 (55.3, 68.3)	0.465	0.575
Preliminary clinical outcomes				
NRS	5.6 (4.6, 6.6)	5.4 (4.9, 5.9)	0.719	0.965
ODI	14.6 (10.9, 18.3)	15.4 (11.4, 19.4)	0.756	0.603

The *p*-values ^†^ were obtained from independent two-sample *t*-tests for comparison between non-thrust and thrust CMT groups. The *p*-values ^‡^ were obtained from Wilcoxon rank-sum tests for comparison between non-thrust and thrust CMT groups. CI, confidence interval; CMT, Chuna manipulative therapy; BMI, body mass index; NRS, Numeric Rating Scale; ODI, Oswestry Disability Index.

**Table 2:** Linear mixed-effects model estimated marginal means, changes from baseline, and between-group differences for NRS and ODI.

Outcomes	Visit	Non-Thrust CMT (Control)			Thrust CMT (Treatment)			Thrust−Non-Thrust		
		EMM (95% CI)	Change from Baseline (95% CI)	*t* (df) (*p*-Value)	EMM (95% CI)	Change from Baseline (95% CI)	*t* (df) (*p*-Value)	MD (95% CI)	Cohen’s *d* (95% CI)	*t* (df) (*p*-Value)
NRS	Baseline	5.6 (5.0, 6.2)			5.6 (5.0, 6.2)			0.0 (−0.8, 0.9)	0.01 (−0.31, 0.33)	0.06 (148.2) (0.954)
	Week 2	5.2 (4.5, 5.8)	−0.4 (−1.4, 0.7)	−0.95 (158.6) (0.801)	5.0 (4.4, 5.6)	−0.5 (−1.6, 0.5)	−1.40 (155.5) (0.530)	−0.1 (−1.0, 0.7)	−0.05 (−0.37, 0.27)	−0.32 (149.9) (0.751)
	Week 3	4.4 (3.8, 5.1)	−1.1 (−2.2, −0.1)	−2.80 (158.6) (0.030)	3.3 (2.7, 3.9)	−2.3 (−3.3, −1.3)	−5.93 (155.5) (<0.001)	−1.2 (−2.0, −0.3)	−0.43 (−0.76, −0.11)	−2.65 (149.9) (0.009)
	Week 4	3.5 (2.9, 4.1)	−2.1 (−3.1, −1.0)	−5.20 (158.6) (<0.001)	2.1 (1.5, 2.7)	−3.5 (−4.5, −2.5)	−8.94 (155.5) (<0.001)	−1.4 (−2.3, −0.5)	−0.51 (−0.84, −0.19)	−3.14 (149.9) (0.002)
	Week 5	3.6 (3.0, 4.2)	−2.0 (−3.0, −0.9)	−4.96 (158.6) (<0.001)	1.3 (0.7, 1.9)	−4.3 (−5.3, −3.3)	−11.04 (155.5) (<0.001)	−2.3 (−3.2, −1.4)	−0.85 (−1.19, −0.52)	−5.22 (149.9) (<0.001)
	Week 12	3.8 (3.2, 4.5)	−1.7 (−2.8, −0.7)	−4.32 (158.6) (<0.001)	1.6 (1.0, 2.2)	−4.0 (−5.0, −2.9)	−9.99 (156.2) (<0.001)	−2.2 (−3.1, −1.3)	−0.80 (−1.13, −0.47)	−4.96 (152.5) (<0.001)
	Week 24	4.0 (3.4, 4.7)	−1.5 (−2.6, −0.5)	−3.80 (158.6) (0.001)	1.6 (1.0, 2.2)	−3.9 (−5.0, −2.9)	−10.11 (155.5) (<0.001)	−2.4 (−3.3, −1.5)	−0.89 (−1.22, −0.55)	−5.43 (149.9) (<0.001)
ODI	Baseline	15.0 (12.7, 17.2)			15.2 (12.9, 17.4)			0.2 (−3.0, 3.4)	0.03 (−0.44, 0.50)	0.12 (69.5) (0.902)
	Week 5	9.8 (7.5, 12.1)	−5.2 (−8.4, −1.9)	−3.63 (52.6) (0.001)	5.5 (3.2, 7.7)	−9.7 (−12.9, −6.5)	−6.94 (51.3) (<0.001)	−4.3 (−7.5, −1.1)	−0.64 (−1.12, −0.16)	−2.69 (70.0) (0.009)
	Week 12	11.6 (9.3, 13.9)	−3.4 (−6.7, −0.1)	−2.39 (52.6) (0.039)	5.2 (2.8, 7.5)	−10.0 (−13.3, −6.8)	−7.02 (52.2) (<0.001)	−6.4 (−9.7, −3.2)	−0.93 (−1.42, −0.44)	−3.92 (70.7) (<0.001)

EMM, estimated marginal mean at the mean baseline value (the two groups share a common model-based baseline EMM by construction); changes from baseline are within-group contrasts vs. the baseline visit (Dunnett-adjusted *p*-values); MD, between-group difference (Thrust−Non-thrust CMT, unadjusted), with negative values favoring thrust CMT. CI, confidence interval; CMT, Chuna manipulative therapy; EMM, estimated marginal mean; MD, mean difference; NRS, Numeric Rating Scale; ODI, Oswestry Disability Index.

**Table 3:** Type III joint tests (ANOVA) of the fixed effects from the linear mixed-effects models (Kenward–Roger degrees of freedom).

Outcome	Term	df1	df2	*F*	*p*-Value
NRS	GROUP	1	25.6	32.40	<0.001
	VISIT	6	156.8	41.19	<0.001
	BASELINE	1	27.2	32.40	<0.001
	SITE	1	26.2	4.31	0.048
	GROUP:VISIT	6	157.4	6.50	<0.001
	VISIT:BASELINE	6	160.1	3.23	0.005
ODI	GROUP	1	25.5	9.99	0.004
	VISIT	2	52.0	33.74	<0.001
	BASELINE	1	25.2	42.65	<0.001
	SITE	1	25.3	10.20	0.004
	GROUP:VISIT	2	51.9	5.64	0.006
	VISIT:BASELINE	2	51.8	18.34	<0.001

F tests for each fixed-effect term (emmeans joint tests, Kenward–Roger denominator degrees of freedom). The GROUP:VISIT row tests the group-by-time interaction. NRS, Numeric Rating Scale; ODI, Oswestry Disability Index.

## Data Availability

The data supporting the findings of this study are not publicly available because they contain individual clinical information that could compromise the privacy of the trial participants, and due to regulatory requirements. Documents related to the clinical trial, including signed protocols, IRB documentation, informed consent forms, source documents, and monitoring records, are securely stored by the principal investigator and clinical trial administrator for a minimum of three years following study completion, in accordance with governmental regulations. These data may be available from the corresponding author upon reasonable request and subject to approval by the institutional authorities.

## Supplementary Materials

The following supporting information can be downloaded at: https://www.mdpi.com/article/10.3390/healthcare14172788/s1, Figure S1: Research process of the study. CMT, Chuna manipulative therapy; F/U, follow-up; Table S1: Schedule of screening, enrollment, intervention, and assessment; Table S2: Representative Chuna manipulative therapy techniques used in each treatment arm; Figure S2: Patient Global Impression of Change (PGIC) at week 5. CMT, Chuna manipulative therapy; PGIC, Patient Global Impression of Change.

## Abbreviations

The following abbreviations are used in this manuscript:

AEs	Adverse events
CI	Confidence intervals
CMT	Chuna manipulative therapy
eCRFs	Electronic case report forms
EQ-5D-5L	5-Level EuroQol 5-dimension
EQ-VAS	EuroQol Visual Analog Scale
FAS	Full analysis set
HVLA	High-velocity, low-amplitude
KMDs	Korean medicine doctors
LBP	Low-back pain
NRS	Numeric Rating Scale
NSAIDs	Nonsteroidal anti-inflammatory drugs
ODI	Oswestry Disability Index
PGIC	Patients’ Global Impression of Change
SAEs	Serious adverse events
SF-12v2	12-Item Short-Form Health Survey
