# Supplementary material for: Thrust Versus Non-Thrust Chuna Manipulative Therapy for Low-Back Pain with Pelvic Deviation: A Multicenter Feasibility Randomized Controlled Trial
Source: Healthcare (Basel). 2026 Sep 1;14(17):2788. doi: 10.3390/healthcare14172788 (PMC13565164; doi:10.3390/healthcare14172788)
Supplement: Supplementary file 1 [file healthcare-14-02788-s001.zip › Table S1..pdf]

Table S1. Schedule of screening, enrollment, intervention, and assessment

Assessment period	Screening visit	Active treatment				Follow-up		
		Week 1	Week 2	Week 3	Week 4	Week 5	Week 12	Week 24
		Assess Visits 1–3	Assess Visits 4–6	Assess Visits 7–9	Assess Visits 10–12	F/U 1	F/U 2	F/U 3
Informed consent form	○							
Body weight	○							
Vital sign check	○	○				○	○	
History (LBP and others, medication)	○							
Diagnostic examination X-ray	○					○		
Confirm suitability for study	○							
Randomization		○						
Credibility and expectancy		○						
Check symptoms and medication change						○	○	○
NRS (low back)	○	○*,**	○**	○**	○**	○	○	○
ODI		○*				○	○	
PGIC						○		
EQ-5D-5L		○*	○**	○**	○**	○	○	○
EQ-VAS		○*	○**	○**	○**	○	○	○
SF-12v2		○*				○	○	
Economic Cost		○*				○	○	

Formatted: Font: (Asian) Malgun Gothic

evaluation	Indicator								
question	iPCQ		○*				○	○	
Check adverse reaction							○	○	○
**Treatment**		**Screening**	**Active treatment**				**Follow-up**		
			**Week 1**	**Week 2**	**Week 3**	**Week 4**	**Week 5**	**Week 12**	**Week 24**
CMT			3-4 times	3-4 times	3-4 times	3-4 times			
Check adverse reactions			Every visit	Every visit	Every visit	Every visit			
Check symptoms and medication change			Every visit	Every visit	Every visit	Every visit			
EQ-5D-5L**, †			△	△	△	△			
EQ-VAS**, †			△	△	△	△			

* Baseline: collected before treatment; ** collected after treatment

† At Visit 1, conduct EQ-5D-5L and EQ-VAS assessments after the treatment; in subsequent visits, perform EQ-5D-5L and EQ-VAS if the participant experiences any adverse events.

CMT, Chuna manipulative therapy; F/U, follow-up; LBP, low-back pain; NRS, Numeric Rating Scale; ODI, Oswestry Disability Index; PGIC, Patients' Global Impression of Change; EQ-5D-5L, Euro Quality of Life-5 dimension; EQ-VAS, Euro Quality of Life-5 visual analogue scale; SF-12v2, 12-Item Short-Form Health Survey; iPCQ, iMTA Productivity Cost Questionnaire.

Formatted: Font: (Asian) Malgun Gothic
