# Supplementary material for: Thrust Versus Non-Thrust Chuna Manipulative Therapy for Low-Back Pain with Pelvic Deviation: A Multicenter Feasibility Randomized Controlled Trial
Source: Healthcare (Basel). 2026 Sep 1;14(17):2788. doi: 10.3390/healthcare14172788 (PMC13565164; doi:10.3390/healthcare14172788)
Supplement: Supplementary file 1 [file healthcare-14-02788-s001.zip › Table S2..pdf]

Table S2. Representative Chuna manipulative therapy techniques used in each treatment arm

Technique	Indication (target deviation)	Patient position	Direction of corrective force
**Non-thrust CMT**			
Iliopsoas myofascial Chuna	Iliopsoas tension or shortening	Supine	Sustained perpendicular pressure at the tender point; the hip is taken to the restrictive barrier and returned to mid-range
Seated lumbar bilateral flexion-malposition muscle relaxation / strengthening	Restricted lumbar extension; bilateral flexion malposition of the lumbar spine	Seated astride the table, trunk extended, arms crossed	Posterior-to-anterior pressure to the extension barrier, against which the patient performs an isometric flexion effort
Quadratus lumborum myofascial Chuna	Quadratus lumborum tension or shortening	Side-lying, ipsilateral arm extended overhead	Separation of the iliac crest from the rib cage, applied on exhalation
Side-lying lumbar oscillatory distraction	General lumbar muscular tension; facet syndrome; lumbar hyperlordosis	Side-lying, deviated side uppermost	Leg drawn posterior-to-anterior and cephalad-to-caudad while the shoulder is pushed anterior-to-posterior, applied rhythmically
Spinal flexion–distraction	General lumbar muscular tension; facet syndrome; lumbar hyperlordosis	Prone on a flexion–distraction table	Longitudinal distraction of the target segment; lateral flexion to the left or right, or circumduction, as indicated
Prone lumbosacral joint distraction	General lumbar muscular tension; facet syndrome; lumbar hyperlordosis	Prone	Cephalad and caudad hands push in opposite directions, distracting the lumbar spine
**Thrust CMT**			
Side-lying lumbar adjustment	Non-neutral extension–rotation–sidebending (ERS) or flexion–rotation–sidebending (FRS) dysfunction	Side-lying, rotated segment downward	Trunk rotated posteriorly while the ilium is drawn antero-superiorly
Supine pubic — superior pubis	Superior pubic displacement	Supine, affected leg off the edge of the table	Superior-to-inferior
Supine pubic — inferior pubis	Inferior pubic displacement	Supine, affected hip and knee maximally flexed	Inferior-to-superior at the ischium, with counter-pressure at the ipsilateral ASIS
Prone iliac — posterior rotation (leg-raise)	Posterior iliac rotation	Prone	Posterior-to-anterior, angled ≈15° laterally from the midline, following the plane of the sacroiliac joint
Prone iliac — anterior rotation	Anterior iliac rotation	Prone	Posterior-to-anterior, slightly lateral-to-cephalad
Prone iliac — inflare / outflare	Inflare or outflare of the ilium	Prone	Inflare: lateral-to-medial. Outflare: medial-to-lateral (supporting hand in

Technique	Indication (target deviation)	Patient position	Direction of corrective force
			the opposite direction)
Prone iliac — posterior rotation with sacral sidebending	Posterior iliac rotation with sacral sidebending	Prone	Two-stage: lateral-to-cephalad with posterior-to-anterior, followed by posterior-to-anterior
Prone sacral — flexion deviation	Sacral flexion deviation	Prone	Posterior-to-anterior, slightly cephalad-to-caudad
Prone sacral — extension deviation	Sacral extension deviation	Prone	Posterior-to-anterior, slightly caudad-to-cephalad
Prone sacral — sidebending with rotation	Left sidebending with right rotation, or right sidebending with left rotation, of the sacrum	Prone	Medial-to-lateral, with the supporting hand lateral-to-medial

Non-thrust techniques were applied within the physiological range of joint motion, and no HVLA technique was applied. In the thrust arm, non-thrust preparatory techniques were applied first to relax the target region, after which the HVLA technique was selected by the treating Korean medicine doctor according to the pelvic deviation pattern identified at diagnosis.

The techniques listed above are those specified in the trial protocol as representative of each arm; in accordance with the protocol, additional techniques within the same category could be applied at the treating doctor's discretion where clinically indicated. Detailed contact points, hand placements, and repetitions for each technique were specified in the trial SOP, and the techniques actually performed were recorded in the electronic case report form at every session. Technique names are English renderings of the Korean terms used in the trial SOP, which follows the standard Chuna manual medicine textbook [12].

ASIS, anterior superior iliac spine; CMT, Chuna manipulative therapy; ERS, extension–rotation–sidebending; FRS, flexion–rotation–sidebending; HVLA, high-velocity, low-amplitude; SOP, standard operating procedure.
